# Novel chemogenetic approaches in modulating calcium signaling

**DOI:** 10.1042/BST20250509

**Published:** 2026-10-28

**Authors:** Anna Price, Tien-Hung Lan, Yubin Zhou

**Affiliations:** 1 Center for Translational Cancer Research, Institute of Biosciences and Technology, College of Medicine, Texas A&M University, Houston, TX 77030, U.S.A.;; 2 Department of Translational Medical Sciences, College of Medicine, Texas A&M University, TX 77030, U.S.A.

## Abstract

Calcium (Ca^2+^) signals regulate diverse processes ranging from neurotransmission and muscle contraction to gene expression, immune response, and cell death. Conventional pharmacological agents are readily applied but often lack molecular and cell-type specificity, whereas genetic perturbations offer greater precision but are typically slow and difficult to reverse. Chemogenetic systems bridge these approaches by combining genetically encoded protein actuators with exogenous chemical ligands. The present review summarizes emerging chemogenetic strategies that modulate Ca^2+^ signaling at distinct steps of the signaling cascade. These include engineered G protein-coupled receptors, chemically inducible receptor tyrosine kinases, direct actuators and inhibitors of STIM–ORAI coupling, genetically encoded blockers of voltage-gated Ca^2+^ channels, and engineered ligand-gated ion channels. We also discuss prospective designs targeting the phospholipase C signaling axis. Collectively, these approaches enable reversible and cell-specific modulation of Ca^2+^ dynamics.

## Introduction

Calcium (Ca^2+^) serves as a ubiquitous second messenger that coordinates a diverse array of cellular processes, including gene transcription, proliferation, migration, immune activation, neurotransmission, and cell death. Intracellular Ca^2+^ concentrations fluctuate dynamically in response to extracellular and intracellular stimuli, generating signals that vary in amplitude, duration, frequency, and subcellular localization. These dynamics arise from coordinated Ca^2+^ release from intracellular stores, influx across the plasma membrane (PM), cytosolic buffering, sequestration into organelles, and extrusion from the cell. Receptors, ion channels, transporters, pumps, and Ca^2+^-binding proteins distributed across the PM, organelle membranes, and the cytoplasm collectively shape the spatial and temporal characteristics of these signals. Precise regulation of Ca^2+^ dynamics is essential for cellular homeostasis, whereas its disruption contributes to numerous pathological conditions [1,2].

Store-operated calcium entry (SOCE) represents a major pathway for Ca^2+^ influx in non-excitable cells. This pathway relies on stromal interaction molecule (STIM) proteins in the endoplasmic reticulum (ER) membrane and ORAI channels in the PM, which together form the Ca^2+^ release-activated Ca^2+^ (CRAC) channel. Following receptor-induced ER Ca^2+^ depletion through inositol 1,4,5-trisphosphate receptor (IP_3_R), STIM1 (or its isoform STIM2) undergoes massive conformational rearrangement that exposes its STIM–ORAI activating region (SOAR). Activated STIM proteins then oligomerize and accumulate at ER–PM junctions, where they directly engage and open ORAI channels for sustained Ca^2+^ influx. The resulting Ca^2+^ signals activate downstream effectors and promote the nuclear translocation of transcription factors such as NF-κB and nuclear factor of activated T cells (NFAT), thereby regulating processes ranging from cell motility and secretion to metabolism, proliferation, gene transcription, and immune-cell activation (Figure 1) [3–5].

In excitable cells, Ca^2+^ influx is mediated primarily by voltage-gated calcium (CaV) channels and ligand-gated channels (also known as ionotropic receptors). CaV channels open in response to membrane depolarization. CaV1 and CaV2 channels are generally activated at relatively positive membrane potentials and exhibit subtype-dependent inactivation kinetics, whereas CaV3 channels activate at lower voltages and inactivate rapidly. By contrast, ligand-gated ion channels open upon binding extracellular ligands, such as neurotransmitters or nucleotides. Depending on their ion selectivity and reversal potential, their activation can depolarize or hyperpolarize the membrane and, in some cases, directly elevate cytosolic Ca^2+^ levels. Major examples include NMDA receptors, Ca^2+^-permeable AMPA receptors, nicotinic acetylcholine receptors, and ATP-gated P2X receptors. Together, these channels generate rapid and spatially restricted Ca^2+^ signals that couple membrane depolarization or ligand recognition to downstream processes such as neurotransmitter release, synaptic plasticity, muscle contraction, hormone secretion, nociception, vascular regulation, and inflammatory signaling [6–8].

Given the central role of Ca^2+^ signaling in physiology and disease, enormous efforts have been devoted to the development of tools for manipulating intracellular Ca^2+^ dynamics. Pharmacological agents remain the most established strategy because they are readily administered, dose-dependent, and often reversible. Clinically approved Ca^2+^-channel blockers, including verapamil, diltiazem, and nifedipine, inhibit CaV channels and are widely used to treat hypertension, angina, and cardiac arrhythmias. Small-molecule inhibitors such as 2-aminoethoxydiphenyl borate, SKF96365, and xestospongin C have also been extensively used to interrogate IP_3_R-mediated Ca^2+^ release and SOCE. However, many of these compounds act on multiple ion channels and signaling proteins, complicating mechanistic interpretation and increasing the risk of off-target effects. Systemic administration also offers limited spatial, temporal, and cell-type specificity, restricting the ability of these agents to manipulate defined Ca^2+^ pathways with precision [9–11].

On the other hand, genetic perturbation approaches, including gene knockout, RNA interference, and CRISPR-mediated genome editing, enable individual components of Ca^2+^ signaling pathways to be examined with greater molecular and cell-type specificity. Nevertheless, conventional genetic perturbations are generally slow, difficult to reverse, and incapable of reproducing the rapid and dynamic nature of Ca^2+^ signals. Sustained loss or overexpression of a signaling component may also induce compensatory changes that obscure its immediate function. Optogenetic technologies address several of these limitations by using light-responsive proteins to control ion channels, receptors, or components involved in Ca^2+^ signaling with high spatial and temporal precision. Their broader application, however, can be constrained by the requirement of specialized illumination hardware, limited penetration of visible light into deep tissues, and potential phototoxicity during prolonged stimulation [12,13].

Chemogenetic approaches provide a complementary strategy for controlling Ca^2+^ signaling. By restricting expression of engineered components to selected cell populations and using small-molecule ligands to control their activity, these systems enable cell type-specific, reversible, and dose-dependent modulation of Ca^2+^ signaling in intact tissues. Chemogenetic tools have been harnessed to regulate receptors, ion channels, intracellular signaling components, or downstream Ca^2+^-responsive effectors, allowing either activation or inhibition of Ca^2+^ dynamics at multiple levels of the signaling cascade. The following sections discuss recent advances in chemogenetic strategies for modulating Ca^2+^ signaling, with emphasis on their mechanisms, applications, advantages, and limitations.

## Chemogenetic control of SOCE

SOCE links extracellular receptor activation to sustained Ca^2+^ influx and is particularly important in non-excitable cells. Activation of GPCRs or RTKs stimulates PLCβ or PLCγ, respectively, leading to the hydrolysis of PIP_2_ into IP_3_ and diacylglycerol (DAG). IP_3_ subsequently binds to IP3Rs in the ER membrane and triggers Ca^2+^ release from the ER lumen into the cytosol. The resulting ER Ca^2+^ depletion activates STIM1, which accumulates at ER–PM junctions and opens ORAI channels to promote Ca^2+^ influx [1,3,11]. Because SOCE involves multiple steps, from GPCRs and RTKs to PLC, IP_3_, STIM1, and ORAI, it provides several molecular entry points for chemogenetic control.

### GPCR-based control

Receptors activated solely by synthetic ligands (RASSLs) were among the earliest engineered GPCRs developed to uncouple receptor activation from endogenous ligand binding. In this strategy, mutations within the orthosteric binding pocket reconfigure ligand recognition, markedly reducing affinity for the endogenous agonist while favoring binding of a synthetic ligand. The first RASSLs were derived from the Gi/o-coupled κ-opioid receptor, and subsequent variants were developed to engage Gs-, Gi/o-, and Gq-dependent signaling pathways (Figure 2A) [14–16]. Reported in 2005, the F435A mutant of the human histamine H1 receptor represented the first Gq-coupled RASSL [17]. This mutation reduced the potency of histamine by approximately 200-fold while increasing the potency of the synthetic agonist 2-phenylhistamine by up to 2600-fold, thereby providing orthogonal control of the Gq-dependent signaling and intracellular Ca^2+^ mobilization [17]. However, broader application of RASSLs has been limited by constitutive receptor activity, residual sensitivity to endogenous ligands, off-target effects of synthetic ligands on wild-type receptors, and pathological consequences associated with receptor overexpression.

Designer receptors exclusively activated by designer drugs (DREADDs) were subsequently developed as an optimized class of RASSL-like GPCRs through directed molecular evolution. The original muscarinic DREADDs, reported in 2007, were engineered to respond to the otherwise pharmacologically inert ligand clozapine-N-oxide (CNO) while showing undetectable activation by acetylcholine [18]. Among these, the Gq-coupled hM3Dq receptor was generated from the human M3 muscarinic receptor by introducing the Y149C and A239G substitutions, enabling CNO-induced activation of Gq signaling, intracellular Ca^2+^ release, and following Ca^2+^ influx through CRAC channels. Related hM1Dq and hM5Dq receptors provide additional muscarinic scaffolds for activating the same Gq-dependent signaling axis, with hM3Dq remaining the most widely used DREADD for chemogenetic control of Ca^2+^ signaling (Figure 2A) [18,19].

The expression of hM3Dq can be restricted using cell type-specific promoters, viral vectors, or recombinase-dependent alleles, thereby enabling remote control of Ca^2+^ signaling in defined cell populations. In neuroscience, hM3Dq has been extensively used to increase neuronal excitability and establish causal relationships between specific neuronal circuits and physiological or behavioral processes, such as feeding, locomotion, reward, sleep, pain, memory, and stress [20]. Beyond the nervous system, Gq-coupled DREADDs have also been expressed in astrocytes, pancreatic β cells, renal epithelia, cardiomyocytes, and other tissues to investigate the role of Ca^2+^ signaling in secretion, metabolism, transport, and tissue remodeling [21–23]. Despite this versatility, DREADD-induced responses do not necessarily recapitulate those initiated by endogenous receptors. Receptor expression levels, prolonged agonist exposure, and concurrent activation of G protein- and β-arrestin-dependent pathways can alter the amplitude, spatial distribution, and kinetics of the resulting Ca^2+^ dynamics. Interpretation is further complicated by the *in vivo* conversion of CNO to clozapine, which can act on endogenous receptors [24]. More potent actuators, including compound 21 and deschloroclozapine, have been developed to activate DREADDs at lower doses and with improved temporal precision [25,26].

Coordination-based chemogenetics provides a distinct route in which an artificial metal-binding site is introduced into a GPCR without disrupting its native ligand-binding mechanism. In the original on-cell coordination chemistry approach (later termed allosteric activation via coordination-based chemogenetics), two histidine residues were incorporated in the vicinity of the ligand-binding pockets of the Gq-coupled mGlu1. Binding of Pd(2,2′-bipyridine), or Pd(bpy), coordinated the engineered residues and stabilized the receptor in an active conformation, thereby activating the downstream Gq signaling pathway and elevating cytosolic Ca^2+^ (Figure 2A). Some double-histidine variants, including mGlu1(P58H/N264H), exhibited both direct agonist activity and positive allosteric modulation of glutamate responses [27].

This approach was refined in 2022, when a single N264H substitution was shown to permit direct activation of mGlu1 by Pd(bpy) without measurably altering glutamate affinity or disrupting receptor native expression and function [28]. A hydrophilic derivative, Pd(sulfo-bpy), reduced nonspecific cellular effects and activated mGlu1(N264H) in acute cerebellar slices. The introduction of N264H into the endogenous mouse Grm1 locus enabled palladium-dependent activation of mGlu1(N264H) expressed at physiological levels, leading to the induction of cerebellar long-term depression. AAV-mediated expression further allowed selective activation of mGlu1(N264H) in Purkinje cells or molecular-layer interneurons, revealing cell-specific effects on synaptic plasticity and neuronal excitability. Importantly, under optimized conditions, the palladium complexes did not activate wild-type mGlu1, supporting the orthogonality of the engineered receptor–ligand pair [28].

Together, RASSLs, DREADDs, and coordination-engineered mGlu1 illustrate complementary design strategies. RASSLs and DREADDs reprogram orthosteric ligand recognition, whereas coordination chemogenetics installs an artificial allosteric site while preserving native agonist responsiveness. None of these receptors modulate Ca^2+^ dynamics directly. Their effects on Ca^2+^ influx depend on the coupling of receptor-driven ER release to SOCE or membrane depolarization, which should be verified in each cellular context.

### RTK-based control

RTKs provide another receptor-based route for chemogenetic modulation of Ca^2+^ signaling. Ligand-induced receptor dimerization promotes trans-autophosphorylation and recruitment of downstream effectors, including PLCγ, which generates IP_3_ and triggers Ca^2+^ release from the ER (Figure 1). This proximity requirement was recapitulated in an early inducible FGFR1 system (iFGFR1), in which the extracellular and transmembrane regions of FGFR1 were replaced by an N-terminal myristoylation sequence, and its intracellular kinase domain was fused to two tandem FKBP(F36V) modules [29]. Addition of the bivalent synthetic dimerizer AP20187 induced receptor oligomerization and phosphorylation, thereby activating downstream MAPK and Akt signaling in cultured cells and driving progressive mammary epithelial transformation in transgenic mice (Figure 2B). More recently, FRB−FKBP heterodimerization was combined with the synthetic multimerization domains HOTag3 and HOTag6 to provide greater control over RTK clustering valency and geometry [30]. In the optimized configuration, membrane-anchored FGFR1−FKBP−HOTag6 was paired with cytosolic FRB-HOTag3, allowing rapamycin-induced higher-order FGFR1 clustering and tunable ERK activation with minimal basal signaling (Figure 2B). Although these studies did not directly examine PLCγ-dependent Ca^2+^ signaling, they established a general strategy for ligand-independent control of RTK intracellular domains.

Caffeine-responsive proximity systems provide a more direct demonstration of chemogenetic control of RTK-based Ca^2+^ signaling. The caffeine-operated synthetic module (COSMO), engineered from an anti-caffeine nanobody, undergoes caffeine-dependent homodimerization [31]. Fusion of COSMO to the intracellular domain of FGFR1 mimicked receptor dimerization and activated PLCγ, cytosolic Ca^2+^ signaling, and the MAPK–ERK pathway (Figure 2B) [31]. However, PM-tethered COSMO-RTK intracellular domains exhibited constitutive signaling in the absence of caffeine, potentially due to the accumulation of lipid-anchored RTK intracellular domains within membrane microdomains, where their high local density may promote spontaneous clustering and kinase activation. To overcome this limitation, COSMO was further evolved into the caffeine-induced heterodimerization system CHASER by inserting a bivalent COSMO module into the anti-mCherry nanobody LaM8 [32]. In the resulting TrkA design, CHASER-TrkA intracellular domain fusion remained cytosolic until caffeine induced its recruitment to tetrameric mCherry anchored at the PM. This recruitment promoted TrkA oligomerization and activated both PLCγ–Ca^2+^ and MAPK–ERK signaling while maintaining minimal basal activity (Figure 2B) [32]. The SAMBA system extends this strategy to a second chemically inducible proximity platform, where salicylic acid, or aspirin following hydrolysis, induces association of the SAMBA-N and SAMBA-C components (Figure 2B) [33]. Fusion of these modules to the TrkA intracellular domain enabled salicylic acid-dependent TrkA activation, resulting in cytosolic Ca^2+^ elevation, NFAT nuclear translocation, and ERK signaling.

These systems show how chemically induced proximity can control receptor activation and downstream Ca^2+^ signaling without engineering the receptor catalytic site. Their modularity also permits the same chemical input to be coupled to different RTKs. Nevertheless, dimerization geometry, receptor expression, basal association, and endogenous actions of the chemical inducer can all affect the response.

### Direct control of STIM–ORAI coupling

Receptor-level chemogenetic tools preserve much of the native signaling cascade but can simultaneously engage multiple downstream branches. Direct manipulation of the STIM–ORAI interaction instead bypasses upstream receptor signaling and provides more selective control over Ca^2+^ influx through CRAC channels. An early demonstration used paired FKBP- and FRB-containing STIM1 chimeras in which the luminal Ca^2+^-sensing EF-SAM domain was replaced with chemically inducible association modules [34]. Addition of the rapalog AP21967 induced STIM1 oligomerization, accumulation at ER–PM junctions, and activation of endogenous CRAC channels without depleting ER Ca^2+^ stores. These experiments established STIM1 oligomerization as a key transduction step linking store depletion to ORAI activation and provided a proof-of-concept for direct chemogenetic control of SOCE.

More recently, truncated STIM1 cytosolic domains have been coupled to chemically induced proximity modules to activate endogenous ORAI channels. In Caf-CRAC, fusion of COSMO to STIM1 residues 233–685 enabled caffeine-induced homodimerization, triggering ORAI-dependent Ca^2+^ influx (Figure 2C) [31]. COSMO was later replaced with the heterodimeric CHASER system, in which caffeine induced association between CHASER-STIM1ct and mCherry-tagged STIM1ct, relieving STIM1 autoinhibition and activating Ca^2+^ influx and NFAT signaling [32]. SAMBA provides a related salicylate-responsive design, where association of SAMBA-N-STIM1ct with multivalent SAMBA-C partners produces valency-dependent CRAC channel activation [33] (Figure 2C). Collectively, these systems directly engage the STIM1–ORAI axis without receptor stimulation, PLC activation, IP_3_ production, or ER Ca^2+^ depletion.

Chemogenetic inhibition of the STIM–ORAI interaction has recently been achieved using CRAC channel inhibitory binders (CRABs) [35]. Derived from the STIM1-binding region of the ORAI3 C terminus, CRABs function as competitive decoys that intercept activated STIM proteins before they engage endogenous ORAI channels. To enable ligand-dependent control, CRAB was fused to an FRB–FKBP module to generate Chemo-CRAB. Rapamycin-induced higher-order oligomerization increased CRAB avidity for the exposed SOAR/CAD domain of activated STIM1, disrupted STIM1–ORAI coupling, and rapidly suppressed both the initiation and maintenance of SOCE (Figure 2C). An alternative Chemo-CRAB design replaced the FRB–FKBP module with a tetrameric FKBP(F36V) cassette responsive to the bivalent synthetic dimerizer AP20187, thereby reducing reliance on immunosuppressive rapamycin. Chemo-CRAB inhibited Ca^2+^ influx driven by Caf-CRAC and Opto-CRAC, as well as Ca^2+^/NFAT signaling downstream of GPCRs, RTKs, and CAR-T-cell activation. It also restored thrombocyte progenitor production in a zebrafish model expressing the constitutively active STIM1 R304W mutant [35].

Direct manipulation of STIM1–ORAI coupling offers greater molecular specificity than receptor-level approaches because it avoids perturbing upstream receptor, PLC, and IP_3_ signaling. This specificity, however, does not necessarily translate into a narrowly confined cellular effect, because STIM1–ORAI coupling serves as a common convergence point for many receptor pathways, and its manipulation may therefore broadly alter SOCE within the engineered cell. Moreover, actuators based on STIM1 fragments bypass luminal Ca^2+^ sensing and may not recapitulate the stoichiometry, ER–PM junctional organization, or feedback regulation of physiological store-operated activation. Receptor-based and channel-based tools thus provide complementary levels of control, with the former preserves more of the native signaling cascade, whereas the latter impose a defined Ca^2+^ input or selectively interrupt CRAC channel activation.

## Chemogenetic control of voltage-gated Ca^2+^ channels

CaV channels couple membrane depolarization to Ca^2+^ influx, but achieving both subtype- and cell type-specific pharmacological control remains challenging because of their conserved channel architecture and overlapping tissue distribution. Chemogenetic strategies can instead exploit intracellular regulatory mechanisms without modifying the ion-conducting pore. An early example, termed GEMIICCs, was developed from Rem, an RGK-family GTPase that potently inhibits high-voltage-activated CaV1 and CaV2 channels [36]. Deletion of the polybasic C terminus of Rem abolished its PM localization and channel-inhibitory activity, leaving the truncated protein (Rem_1–265_) predominantly cytosolic. Fusion of Rem_1–265_ to the C1 domain of protein kinase Cγ restored PM targeting in response to phorbol-12,13-dibutyrate (PdBu), resulting in rapid inhibition of recombinant CaV1.2 and CaV2.2 currents. A parallel GEMIICC design using rapamycin-induced heterodimerization between FKBP-tagged Rem_1–265_ and a constitutively PM-targeted FRB domain achieved similar inducible channel inhibition (Figure 3A).

The channel inactivation induced by membrane tethering of an associated protein (ChIMP) strategy generalized this strategy beyond RGK proteins [37]. Channel-associated proteins that bind intracellular regions of CaV α1 subunit, including CaVβ, 14-3-3, calmodulin, and a catalytically inactive CaMKII fragment, were converted into blockers through inducible or constitutive PM targeting. Because these proteins engage different channel interfaces, the resulting inhibitors exhibited different subtype selectivity, kinetics, and potency.

More recently, the endogenous C-terminus-mediated inhibition (CMI) mechanism of L-type CaV1 channels was adapted for chemogenetic control [38]. CaV1.3-derived proximal and distal C-terminal regulatory domains (PCRD and DCRD) were fused to FKBP and recruited by rapamycin to a constitutively PM-anchored FRB-Ras module (Figure 3A). This membrane recruitment brought the inhibitory peptides into proximity with the channel, leading to acute inhibition of both short and long CaV1.3 variants, suppressed spontaneous Ca^2+^ activity in cortical neurons, and disruption of the coupling between CaV1-dependent Ca^2+^ influx–neuritogenesis [38].

## Chemogenetic control of ligand-gated ion channels

The PSAM/PSEM system was developed by re-engineering the ligand-binding domain of the α7 nicotinic acetylcholine receptor (α7 nAChR) to recognize synthetic PSEMs while reducing responsiveness to acetylcholine [39]. This transferable ligand-binding domain can be combined with ion-pore domains from different Cys-loop receptors to generate channels with distinct ionic conductances. For direct control of Ca^2+^ influx, PSAM (Q79G, L141S) was coupled to the predominantly Ca^2+^-permeable α7 nAChR pore and further modified with the V13′T (V274T) gating mutation. The resulting channel showed minimal desensitization and supported sustained PSEM^9S^-dependent Ca^2+^ influx (Figure 3B) [39]. By contrast, fusion of PSAMs to the cation-selective 5-HT3 receptor pore generated depolarizing currents and increased neuronal firing, which can secondarily promote Ca^2+^ entry through endogenous CaV channels. Fusion to the chloride-selective glycine receptor pore instead reduced input resistance and neuronal excitability, thereby indirectly limiting activity-dependent Ca^2+^ influx. The later PSAM^4^ system was optimized for activation by low-nanomolar concentrations of varenicline [40]. PSAM^4^-GlyR enabled reversible neuronal silencing following low-dose varenicline administration in mice and was further validated electrophysiologically in the globus pallidus of a rhesus monkey. Varenicline analogs, including uPSEM792 and uPSEM817, subsequently achieved subnanomolar potency at PSAM^4^ channels together with substantially improved selectivity over endogenous α7 and α4β2 nicotinic acetylcholine receptors and 5-HT3 receptors [40].

The bradanicline- and acetylcholine-activated receptor for neuronal inhibition (BARNI) extends this inhibitory Cys-loop receptor architecture while deliberately retaining responsiveness to an endogenous ligand [41]. The BARNI combines a modified α7 nAChR ligand-binding domain with the chloride-permeable pore domain of the α1 glycine receptor. In hippocampal neurons, bradanicline-induced activation of BARNI reduced input resistance, increased the current required to evoke action potentials, and suppressed neuronal firing. *In vivo*, hippocampal expression of BARNI increased the threshold for electrically evoked seizures and reduced spontaneous seizure frequency in a mouse model of chronic epilepsy. Its effects on Ca^2+^ signaling are therefore indirect, arising from reduced neuronal excitability and diminished recruitment of endogenous CaV channels. Because BARNI remains responsive to acetylcholine, endogenous cholinergic signals, including those accompanying seizure activity, may also contribute to receptor activation [41].

Luminopsins (LMOs), or luminescent opsins, provide a hybrid opto-chemogenetic strategy for chemical control of light-gated ion channels. LMOs are genetically encoded fusion proteins composed of a light-emitting luciferase and a light-responsive ion channel, such that administration of a luciferase substrate generates bioluminescence that activates the tethered opsin [42,43]. Early excitatory LMOs combined *Gaussia princeps* luciferase (GLuc) with channelrhodopsin-2 (ChR2) from *Chlamydomonas reinhardtii* or the more red-shifted excitation spectrum Volvox channelrhodopsin-1 from *Volvox carteri*. Oxidation of coelenterazine by GLuc activates the channelrhodopsin, producing an inward cation current and membrane depolarization. Subsequent designs improved this bioluminescence-to-opsin coupling by incorporating brighter luciferases, such as the slow-burn GLuc variant used in LMO3, and more recently, engineered luciferase-fluorescent protein emitters paired with highly light-sensitive opsins [43,44]. Because canonical channelrhodopsins exhibit relatively low Ca^2+^ permeability, their effects on intracellular Ca^2+^ often arise predominantly from membrane depolarization and following activation of endogenous CaV channels. Continued engineering on channelrhodopsins has generated variants with substantially enhanced Ca^2+^ permeability, including CatCh, CapChR1, and CapChR2 [45]. Incorporation of such Ca^2+^-permeable opsins into luminopsin architectures could further expand this platform toward more direct control of Ca^2+^ influx.

Ligand-gated chemogenetic channels provide rapid, receptor-based control of electrical activity and Ca^2+^ signaling, but a cationic current should not automatically be equated with direct Ca^2+^ influx. The magnitude and direction of the resulting response depend on pore selectivity, reversal potential, resting membrane potential, endogenous conductances, and intracellular chloride concentration. Accordingly, Ca^2+^ imaging should be combined with electrophysiology and, where appropriate, ion-substitution or selective channel-blockade experiments to distinguish direct permeation through the engineered channel from secondary activation or inhibition of endogenous CaV channels.

## Potential engineering targets and design opportunities

Most established chemogenetic systems regulate receptors or channels, but the PLC–PIP_2_–IP_3_ cascade offers several additional engineering targets for controlling Ca^2+^ signaling. Recent optogenetic studies demonstrate that PLC enzymes themselves can be engineered as direct actuators, providing useful starting points for future chemogenetic designs. Opto-PLCβ used light-dependent PM targeting to trigger reversible and spatially restricted PIP_2_ hydrolysis, DAG production, and Ca^2+^ signaling [46]. Similarly, light-induced PM recruitment of cancer-associated PLCγ1 variants, including P867R, S345F, and D1165H, activated lipid hydrolysis [47]. Together, these findings suggest that chemogenetic PLC actuators could be developed by replacing light-dependent recruitment with chemically induced proximity. Direct control of PLC would bypass upstream receptors but would not selectively regulate SOCE, because PLC activation simultaneously depletes PIP_2_ and generates IP_3_ and DAG, thereby engaging Ca^2+^ release, DAG–PKC signaling, and other PIP_2_-dependent processes.

Downstream of PLC, direct sequestration of IP_3_ may represent a more selective target of suppressing Ca^2+^ release. A recombinant ‘IP_3_ sponge’, derived from the ligand-binding domain of mouse IP_3_R1, was engineered to bind IP_3_ with approximately 1000-fold higher affinity than the parental receptor [48]. Constitutive expression of the sponge competitively sequestered IP_3_ and inhibited IP_3_-mediated Ca^2+^ release. Although the original IP_3_ sponge is not chemically switchable, it serves as a well-validated effector that could be incorporated into ligand-dependent oligomerization, localization, or allosteric-control systems. For example, a cytosolic low-affinity or split sponge could be activated or concentrated near IP_3_-producing membranes only after chemically induced assembly.

PIP_2_ availability can likewise be manipulated through chemically induced enzyme recruitment. Rapamycin-induced FKBP–FRB heterodimerization has been used to recruit an inositol 5-phosphatase to the PM, enabling rapid conversion of PIP_2_ to phosphatidylinositol 4-phosphate without directly generating IP_3_, DAG, or Ca^2+^ transients [49]. This approach was initially developed to interrogate the role of PIP_2_ in ion-channel gating and other membrane-associated processes, but it also illustrates how chemogenetic tools can intervene upstream of IP_3_ production. Although acute PIP_2_ depletion can terminate receptor-evoked Ca^2+^ signals, the original studies did not examine SOCE as the functional readout. Whether this strategy can be configured to inhibit SOCE reproducibly and selectively therefore remains to be directly validated. Moreover, its broader utility for selective modulation of Ca^2+^ signaling is limited because PIP_2_ is required for the function of numerous ion channels, transporters, endocytic proteins, cytoskeletal regulators, and signaling complexes. Acute PIP_2_ depletion may consequently exert widespread effects that extend well beyond the PLC–PIP_2_–IP_3_ pathway.

Protein–protein interaction inhibitors may offer a more pathway-selective alternative. A helix-turn-helix peptide derived from PLCβ3 binds the active Gαq at its canonical effector interface and competitively prevents Gαq-dependent activation of PLCβ. An optogenetic implementation, Opto-dHTH, has already demonstrated that light-induced PM targeting of this decoy can reversibly suppress receptor-induced PIP_2_ hydrolysis, DAG production, and Ca^2+^ signaling within selected subcellular regions [50]. In principle, this architecture could be translated into chemogenetic control by replacing the optical recruitment pair with a ligand-induced dimerization module. Similarly, analogous decoys could be designed to block PLCγ recruitment to phosphorylated RTKs through Src homology 2 domain-mediated interactions or to disrupt defined contacts between PLC, membranes, and scaffold proteins [51,52]. Although these designs remain largely prospective in the chemogenetic setting, they illustrate a potential strategy for converting biochemically validated inhibitory peptides into reversible switches controlled by chemically induced proximity.

Future tool development should prioritize tunable and reversible control rather than maximal signal amplitude. Ca^2+^-dependent signaling is determined by signal amplitude, duration, oscillation frequency, as well as subcellular localization, and the same actuator may produce different responses across cell types. Useful benchmarks therefore include basal activity, dose–response relationship, activation and deactivation kinetics, direct versus indirect Ca^2+^ entry, ligand pharmacokinetics, and effects on endogenous targets. Orthogonality should be evaluated at both sides of the pair, with the protein component remaining minimally responsive to native inputs and the chemical exerting minimal effects on endogenous pathways. Combining these measurements with cell-specific expression and disease-relevant models will be essential for moving chemogenetic control of Ca^2+^ signaling from proof-of-concept demonstrations toward mechanistic and therapeutic applications

## Figures and Tables

**Figure 1.**
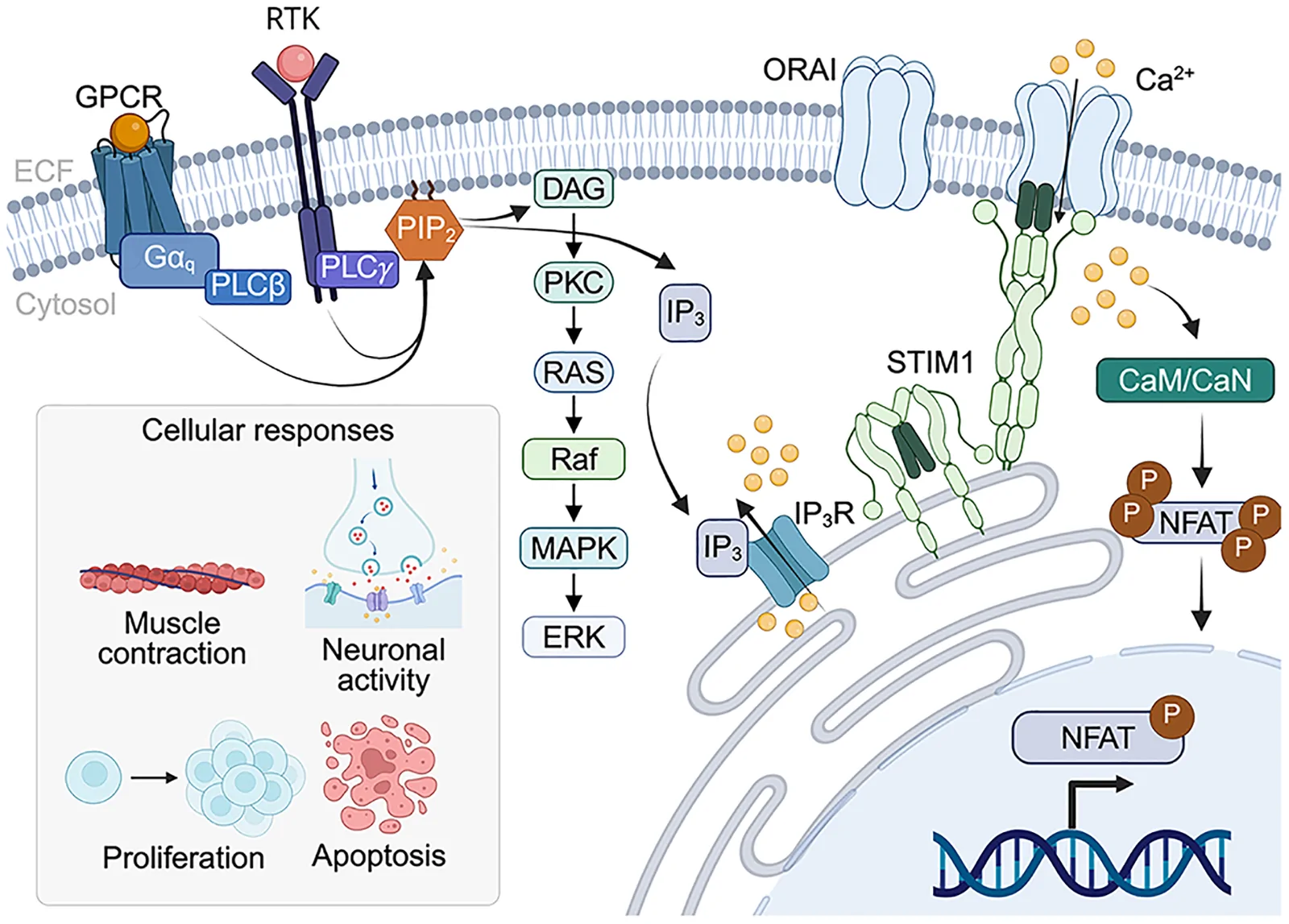
Overview of receptor-medicated Ca^2+^ signaling Extracellular ligand binding to G protein-coupled receptors (GPCRs) or receptor tyrosine kinases (RTKs) activates phospholipase C-β (PLCβ) or phospholipase C-γ (PLCγ), resulting in hydrolysis of phosphatidylinositol 4,5-bisphosphate (PIP_2_) into inositol 1,4,5-trisphosphate (IP_3_) and DAG. IP_3_ binds to IP_3_R on the ER, promoting ER Ca^2+^ release, whereas DAG activates PKC and downstream MAPK/ERK signaling. Depletion of ER Ca^2+^ stores induces conformational changes in STIM1, exposing the SOAR domain. Activated STIM1 accumulates at the ER–PM junctions, where it directly engages and activates ORAI channels to mediate SOCE. Elevated cytosolic Ca^2+^ activates downstream effectors such as CaM, CaN, and NFAT, thereby regulating diverse cellular processes. Created in BioRender. Price, A. (2026) https://BioRender.com/yk1k7gx.

**Figure 2.**
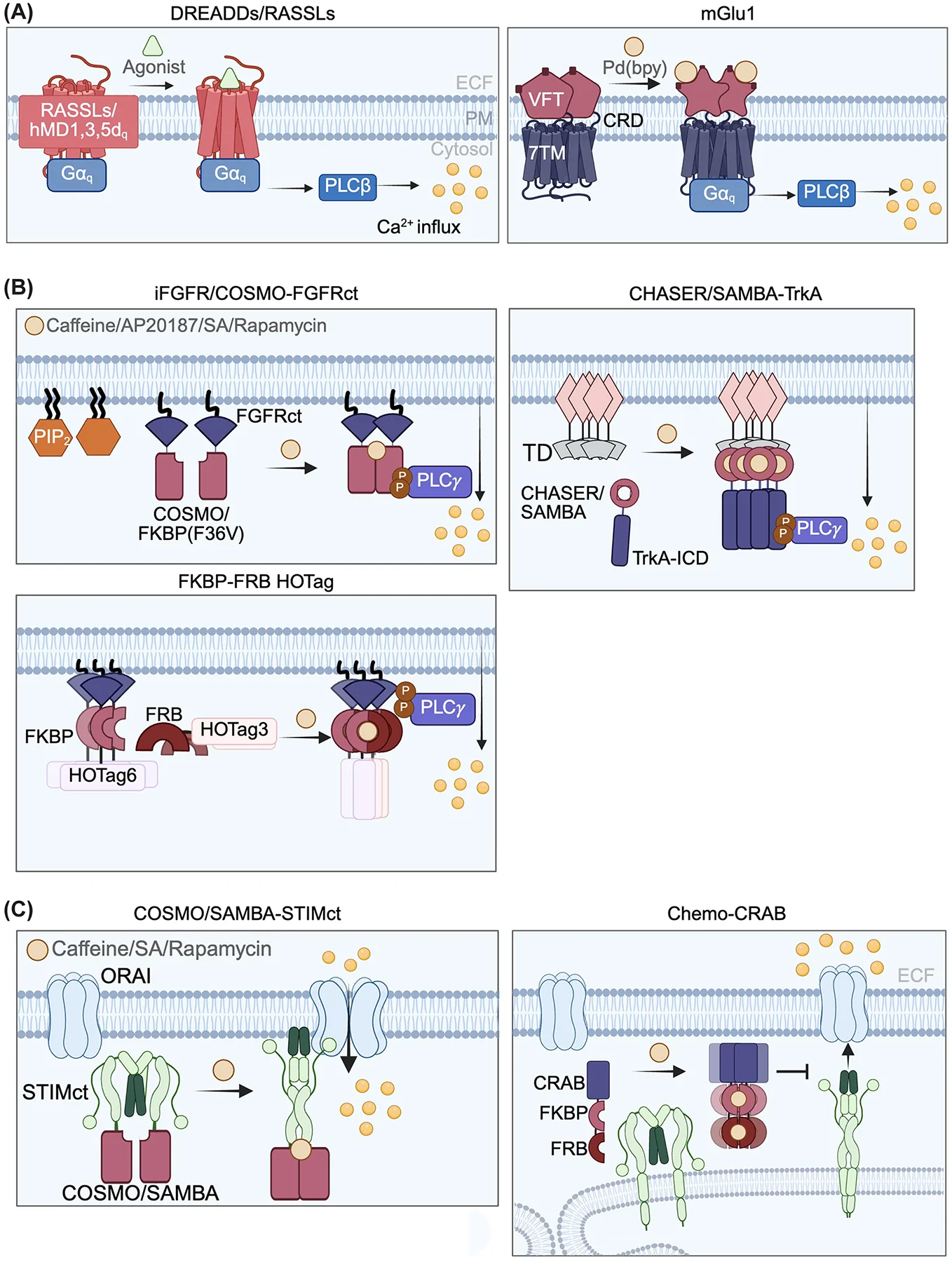
Chemogenetic modulation of SOCE (**A**) GPCR-based indirect control of Ca^2+^ signaling. Left, DREADDS/RASSLs: GPCR ligand specificity is reprogrammed for selective activation by synthetic agonists while minimizing responsiveness to endogenous ligands. RASSLs were generated through mutagenesis of residues within the orthosteric ligand-binding pocket to alter receptor pharmacology. DREADDs were developed through iterative mutagenesis and functional screening to improve ligand selectivity and reduce constitutive activity. Ligand-induced receptor activation stimulates Gq-dependent PLCβ signaling, resulting in IP_3_ production, ER Ca^2+^ release, and subsequent SOCE. Right, Coordination-engineered metabotropic glutamate receptor 1 (mGlu1): Artificial metal-binding site introduced into mGlu1 enables activation by palladium coordination complexes, including Pd(bpy), while preserving native glutamate responsiveness. Metal coordination stabilizes the receptor in its active conformation, activating the Gq–PLCβ signaling pathway and indirectly promoting Ca^2+^ influx. Created in BioRender. Price, A. (2026) https://BioRender.com/1rsm0w7. (**B**) RTK-based indirect control of Ca^2+^ signaling. Left, iFGFR/COSMO-FGFRct: Chemically induced dimerization of the FGFR1 intracellular domain enables ligand-dependent receptor activation. iFGFR1 fused to FKBP(F36V) domains is dimerized by the synthetic ligand AP20187. COSMO-FGFR1ct uses caffeine-induced COSMO homodimerization to promote receptor clustering. Activated FGFR1 stimulates the downstream PLCγ signaling cascade. Right, CHASER/SAMBA-TrkA: CHASER or salicylic acid-mediated binary association (SAMBA) activates the TrkA intracellular domain through caffeine- or salicylic acid-dependent heterodimerization, mimicking NGF-dependent receptor activation and triggering PLCγ-mediated Ca^2+^ signaling. Bottom left, FKBP-FRB-HOTag: Membrane-anchored FKBP recruits the cytosolic FRB-HOTag6-FGFR1 fusion protein to the PM following rapamycin addition. HOTag3/HOTag6 self-assembly promotes receptor clustering and trans-autophosphorylation, activating PLCγ signaling cascade. Created in BioRender. Price, A. (2026) https://BioRender.com/1rsm0w7. (**C**) Direct modulation on STIM1−ORAI1 coupling. Left, COSMO/SAMBA-STIMct: Caffeine- or salicylic acid-dependent dimerization of COSMO- or SAMBA-fused truncated STIM1 cytosolic domains promotes STIM1 oligomerization, enabling direct engagement and activation of endogenous ORAI channels without upstream receptor activation or ER Ca^2+^ store depletion. Right, Chemo-CRAB: CRAB, derived from the STIM1-binding region of the ORAI3 C-terminus, functions as competitive inhibitors by preventing STIM1–ORAI coupling. Rapamycin-induced FKBP–FRB oligomerization increases CRAB avidity toward the exposed SOAR/CAD domain of activated STIM1, thereby suppressing SOCE and downstream Ca^2+^ signaling. Created in BioRender. Price, A. (2026) https://BioRender.com/1rsm0w7.

**Figure 3.**
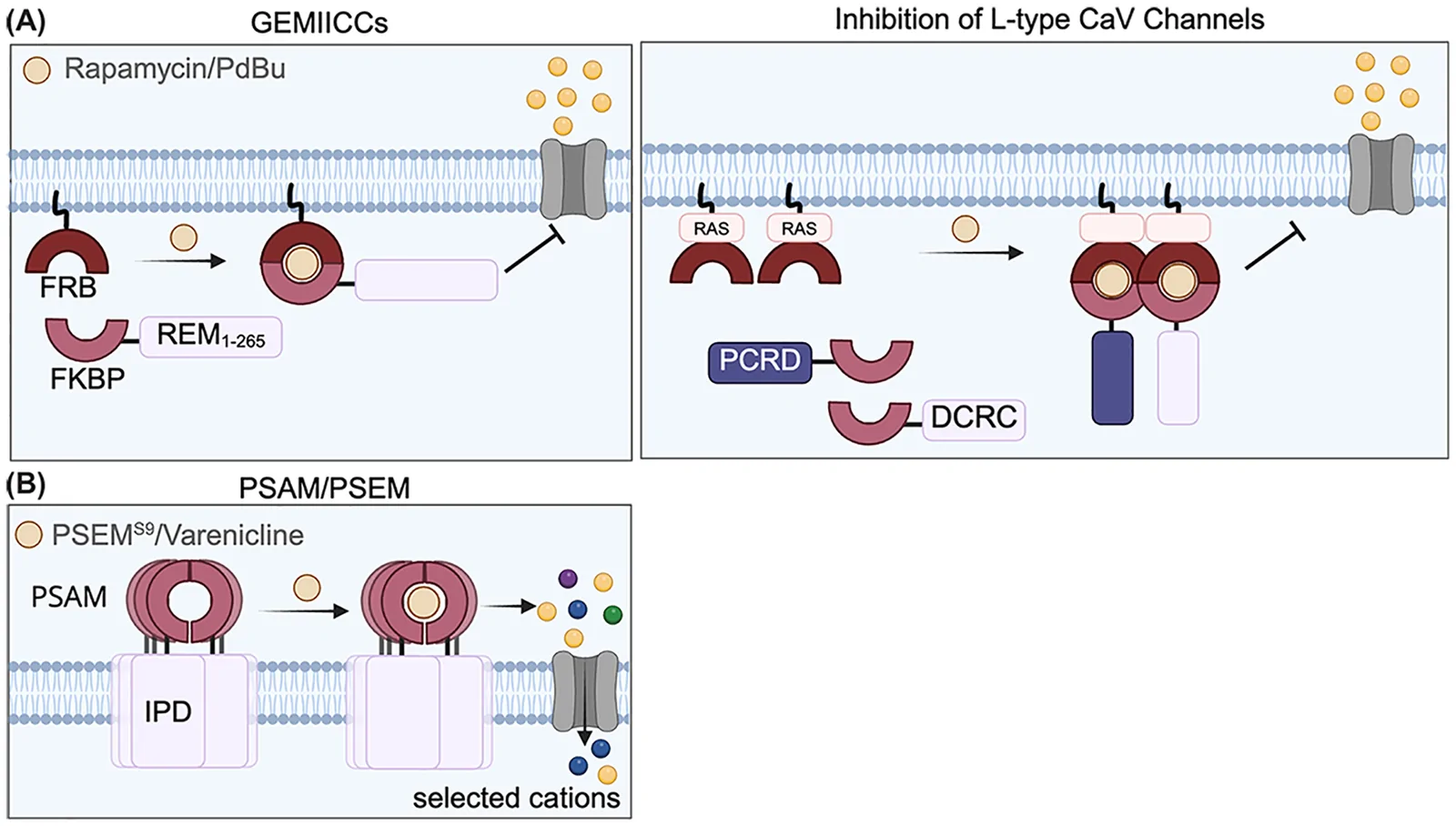
Chemogenetic modulation of ion channels (**A**) Direct chemogenetic inhibition of voltage-gated ion channels. Left, genetically encoded molecules for inducibly inactivating CaV channels (GEMIICCs): RGK family GTPase Rem was repurposed to achieve chemically controlled inhibition of voltage-activated CaV channels. Deletion of the native polybasic C-terminal membrane-targeting sequence renders Rem cytosolic, whereas fusion to FRB enables rapamycin-induced recruitment to membrane-anchored FKBP-Ras. Restored PM localization allows Rem to reassociate with CaV channel complexes, resulting in rapid and reversible inhibition of Ca^2+^ influx. Right, Inhibition of L-type CaV channels: The intrinsic autoinhibitory mechanism of CaV1.3 is adapted for chemogenetic regulation by separating the proximal and distal C-terminal regulatory domains (PCRD and DCRD). PCRD is fused to FKBP and recruited to the membrane-anchored FRB-Ras following rapamycin-induced heterodimerization, reconstituting the interaction with DCRD and restoring CMI, thus suppressing CaV1-dependent Ca^2+^ influx. Created in BioRender. Price, A. (2026) https://BioRender.com/azm0t83. (**B**) Pharmacologically selective actuator module/pharmacologically selective effector molecule (PSAM/PSEM) control of ligand-gated ion channels. PSAMs were developed by engineering the ligand-binding domain of the α7 nicotinic acetylcholine receptor to selectively recognize synthetic PSEMs, while minimizing responsiveness to endogenous acetylcholine. Fusion of the engineered ligand-binding domain to interchangeable Cys-loop receptor pore domains generates channels with distinct ion selectivity. Administration of PSEMs selectively gates the engineered receptors, thereby modulating intracellular Ca^2+^ influx and downstream signaling. Created in BioRender. Price, A. (2026) https://BioRender.com/azm0t83.

## Perspectives

- Ca^2+^ signaling controls diverse physiological functions by encoding information through changes in signal amplitude, duration, frequency, and subcellular localization.
- Current chemogenetic platforms regulate Ca^2+^ signaling at multiple levels, from receptors to ion channels, but their apparent selectivity depends on ligand orthogonality, expression level, and whether Ca^2+^ entry occurs directly or secondarily through store release or membrane depolarization.
- Future systems should minimize basal activity, responsiveness to endogenous ligands, and off-target effects of chemical actuators, with careful consideration of the trade-off between preservation of native signaling with pathway specificity. Chemically gated inhibitory modules targeting the PLC–PIP_2_–IP_3_ axis represent a promising direction.

## Abbreviations

BARNI: bradanicline- and acetylcholine-activated receptor for neuronal inhibition
CaV: voltage-gated calcium
COSMO: caffeine-operated synthetic module
CMI: C-terminus-mediated inhibition
CRABs: CRAC channel inhibitory binders
ChR2: channelrhodopsin-2
CNO: clozapine-N-oxide
DAG: diacylglycerol
DREADDs: designer receptors exclusively activated by designer drugs
ER: endoplasmic reticulum
GEMIICCs: genetically encoded molecules for inducibly inactivating CaV channels
Gluc: *Gaussia princeps* luciferase
GPCRs: G protein-coupled receptors
IP_3_: inositol 1,4,5-trisphosphate
IP_3_R: inositol 1,4,5-trisphosphate receptor
LMOs: luminopsins
mGlu1: metabotropic glutamate receptor 1
NFAT: nuclear factor of activated T cells
α7 nAChR: α7 nicotinic acetylcholine receptor
PIP_2_: phosphatidylinositol 4,5-bisphosphate
PLCβ: phospholipase C-β
PLCγ: phospholipase C-γ
PM: plasma membrane
PSAM: pharmacologically selective actuator module
PSEM: pharmacologically selective effector molecule
RASSLs: receptors activated solely by synthetic ligands
RTKs: receptor tyrosine kinases
SOCE: store-operated calcium entry
SOAR: STIM–ORAI activating region
STIM: stromal interaction molecule
